# Effect of external cephalic version in a resource-limited setting on the Thailand-Myanmar border: a retrospective cohort with propensity score analysis

**DOI:** 10.1186/s12884-026-08917-5

**Published:** 2026-03-12

**Authors:** Nay Win Tun, Nienke Vonk, Aung Myat Min, Mary Ellen Gilder, Gabie Hoogenboom, Lay Lay Wah, Wah Say, François Nosten, Marcus J. Rijken, Rose McGready, Sue J. Lee

**Affiliations:** 1 Shoklo Malaria Research Unit, Mahidol-Oxford Tropical Medicine Research Unit, Faculty of Tropical Medicine, Mahidol University, Mae Ramat, Thailand; 2 Amsterdam University Medical Center, Amsterdam, The Netherlands; 3 Centre for Tropical Medicine and Global Health, Nuffield Department of Medicine, University of Oxford, Oxford, UK; 4 Julius Centre Global Health, Utrecht, The Netherlands; 5 Utrecht University Medical Center, Utrecht, The Netherlands; 6 Mahidol-Oxford Tropical Medicine Research Unit (MORU), Faculty of Tropical Medicine, Mahidol University, 420/6 Rajvithi Road, Bangkok, 10400 Thailand

**Keywords:** Propensity scores, Breech presentation, External cephalic version, Antenatal care, Resource-limited setting

## Abstract

**Background:**

External cephalic version (ECV) is recommended to reduce the risk of breech presentation at birth. This study analysed the effect of external cephalic version (ECV) or not, on breech presentation at birth in a resource-limited setting.

**Methods:**

Women with ultrasound confirmed breech presentation from 28 weeks gestation at antenatal clinics of the Shoklo Malaria Research Unit (SMRU) and a known pregnancy outcome, from 2008 to 2018 along the Thailand-Myanmar border were included. Propensity score analysis using inverse probability weighting compared breech at birth between women who had ECV offered or not. Pregnancy outcomes were compared between ECV successful and unsuccessful versions. Adverse perinatal outcomes included cord prolapse, fetal distress, Apgar < 7 at 5 min, stillbirth and early neonatal death.

**Results:**

Among 504 women with breech presentation between 35–37 weeks, 330 were offered ECV and 174 were not. Breech at birth in women offered ECV was 50.9% (168/330) compared to 47.7% (83/174) in women with no ECV attempt. In other words, the rate of spontaneous version from breech to cephalic in women with no ECV attempt was 52.3% (91/174). Propensity score analysis indicated no association between ECV being offered or not and breech presentation at birth (adjusted Odds Ratio 1.23, 95% confidence interval 0.82–1.83). Caesarean section for breech 22.4% (74/330) vs 20.1% (35/174), *p* = 0.540; and adverse perinatal outcomes, 5.2% (17/330) vs 7.5% (13/174), *p* = 0.295, were similar whether ECV was offered or not.

Among all women offered ECV (*n* = 537, range 32–40 weeks), breech at birth (4.4% (12/273) vs 90.9% (240/264), *p* < 0.001; caesarean section for breech 1.1% (3/273) vs 40.5% (107/264), *p* < 0.001; and adverse perinatal outcomes 3.7% (10/273) vs 9.1% (24/264), *p* = 0.010, were significantly lower in the successful vs unsuccessful groups.

**Conclusion:**

ECV was safely offered in a resource-limited setting. Comparison of ECV offered or not at 35 to 37 weeks suggested no benefit or harm with respect to presentation at birth in contrast to comparison of ECV success or unsuccessful at 32 to 40 weeks. Improving the ECV success rate of health care practitioners, or task shifting, could positively contribute to optimising the potential benefits of ECV.

**Supplementary Information:**

The online version contains supplementary material available at 10.1186/s12884-026-08917-5.

## Background

Breech presentation is associated with higher maternal morbidity, congenital abnormalities, perinatal mortality and morbidity, compared to cephalic presentation. This increased risk is due to multiple factors: preterm birth, physiologically challenging mechanism of birth, and practitioner’s inexperience with vaginal breech birth resulting in birth asphyxia or trauma [1–6]. Limited data from low-income countries (LIC) suggests higher perinatal mortality with vaginal breech birth compared to caesarean breech birth at the cost of higher maternal morbidity and mortality [7].

External Cephalic Version (ECV) is considered non-invasive, safe, and cost-effective and is a recommended medical procedure for the 2–5% of singletons who are breech at 36 to 37 weeks’ gestation or later [8, 9]. The evidence base for management of breech pregnancy is also constrained within randomized trials of ECV [10–13] that are difficult to compare because they vary in their timing of ECV and the number of ECV attempts.

The Royal College of Obstetrics and Gynaecology (RCOG), UK, report ECV success of about 50%; 40% for nulliparous women and 60% for multiparous women, assuming a median gestational age of 40 weeks. Spontaneous reversion back to breech after successful ECV is reported to be less than 5% [9]. Where randomization is not feasible a statistical method called “propensity score analysis” is increasingly being used in observational studies to understand medical interventions, including in obstetric care [14, 15]. The propensity scores use “a causal inference technique for treatment effect estimation in observational studies by accounting for the conditional probability of treatment selection” [16], which is useful because the risk of bias is reduced.

Utilising retrospective antenatal care data can contribute to reducing the significant lack of evidence on breech pregnancy and implementation and success of ECV, in particular in resource-limited settings where this cost-effective procedure could contribute to reducing maternal morbidity and perinatal mortality and morbidity. Data paucity in LIC derives in part from limited ultrasound availability and poor allocation of health personnel skilled in obstetric and newborn care [17, 18]. As a result, there is no evidence on the numbers of sonographers who can make the ultrasound assessment to identify breech and, more importantly, exclude contraindications to ECV, nor are there data on personnel skilled in ECV and their success and adverse events rate.

Our main aim was to determine the effect of attempting external cephalic version (ECV) or not, on breech presentation at birth in a low-income setting that has access to ultrasound (at no cost to the pregnant woman). In this resource-limited setting with sites spread over 150 km it was not possible to always have a doctor with ECV skills available when women were in the gestational window for ECV. A further aim was to compare maternal and perinatal outcomes among women who were offered ECV (i.e., successful or not) and to describe adverse outcomes from ECV attempts.

## Methods

### Study setting

The Shoklo Malaria Research Unit (SMRU) provides a range of basic but quality driven health care initiatives for marginalized populations living on both sides of the Thailand-Myanmar border, in Tak Province, in refugees (1986–2016) and migrants (1998-present). Maternal and child health is one of the focal health care areas and clinics are able to provide free antenatal, intra-partum and post-partum care by Skilled Birth Attendants (SBAs) who are supported by expatriate doctors [19]. Since 2001, estimated Gestational Age (EGA) during antenatal care is obtained predominantly by ultrasound assessment at the first visit regardless of gestational age, and with the aim of routine scans at 8–14 weeks, and 22–24 weeks [20]. Women who attend after 24 weeks have both ultrasound and the neonate age assessment by Dubowitz since the date of their last menstrual period is frequently unavailable [21]. Any woman with a breech presentation from 28 weeks onwards receives repeated ultrasound for confirmation.

The mode of delivery and other details are routinely recorded in the SMRU database which are archived digitally or paper-based e.g. partogram. The birth units are led by a senior SBA (midwife led practice) with a 24 h doctor available but not always physically present; and all delivery room staff are trained in breech vaginal birth using the Advanced Life Support in Obstetrics course [22].

### External cephalic version

The ECV procedure was introduced in 2008 and only attempted when the doctor was available [23]. SMRU ECV procedure is detailed in the SMRU Obstetric Manual (Additional file 1). Women with breech presentation at 35–37 weeks were offered external cephalic version (ECV). ECV was carried out in the ultrasound room. A scan was conducted to exclude placenta praevia and major abnormalities, and to confirm amniotic fluid index (AFI), fetal presentation and heart rate. If there were no major contraindications the woman was counselled about the options. If verbal permission was obtained an emergency car was placed on standby. Doctors were trained to obtain a degree of the Trendelenburg position (i.e., 15–30 degree incline with the feet elevated above the head) by using bricks under the distal legs of the bed, and the use of uterine relaxant (subcutaneous terbutaline) and gel (or dry) was dependent on doctor preference; these factors were not systematically recorded. The breech was disengaged from the pelvis and a gentle forward roll commenced; the procedure continued unless the woman expressed discomfort or doctor did not feel progress. A backward roll was also tried if forward roll failed and the woman was willing to proceed. Fetal heart beat was checked every 15 min for 1 h after ECV or more frequently, if indicated. A successful ECV was defined as a roll that resulted in turning the fetus to the cephalic position.

### Eligible population and inclusion

This was a retrospective population cohort study of singleton pregnancies in women who had fetal presentation by ultrasound recorded at a gestational age of 28 weeks or more with a known birth outcome, between 2008 and 2018. For the propensity score analysis, all records of women with a breech presentation at 35–37 weeks gestation were reviewed independently by two authors to confirm if ECV was done or not done (NWT and RM). For pregnancies with contraindications to ECV, the reason was extracted from the records. For the comparison of maternal and perinatal outcomes, all women with an ECV attempt were included and records were reviewed by two authors (NWT and RM) for success or not.

### Primary outcome

The primary outcome was the proportion of breech at birth.

### Secondary outcomes

Secondary outcomes included adverse outcomes at the time of ECV, and adverse maternal and perinatal outcomes.

### Variables collected

Maternal baseline characteristics included age, parity (0, 1, 2 +), marginalized status (refugee or migrant), literacy (self-reported ability to read yes/no), smoking (yes/no), and estimated gestational age at first antenatal care (ANC) visit (first ANC in trimester 3 yes/no). Maternal characteristics during pregnancy included pre-eclampsia, date and fetal presentation (every recorded visit from 28 weeks) and fetal presentation at labour, and after delivery placental abruption.

ECV detail included the year, placental position, EGA at ECV, body mass index (BMI) at ECV, ECV contraindication, ECV procedural outcome (successful/unsuccessful), complications from ECV, and the total number of visits where ECV was attempted (range: 1–3 visits).

Outcome of delivery included type of birth (breech at birth, including vaginal breech birth and caesarean breech birth; cephalic vaginal birth, assisted cephalic vaginal delivery and cephalic caesarean birth; and place of birth (SMRU, home or hospital).

Neonatal outcomes included sex, estimated gestational age at birth (preterm < 37.0 weeks), birthweight (measured within 72 h of birth), congenital abnormality (newborn with abnormalities identified by surface examination and cardiac auscultation), and adverse perinatal outcomes including: cord prolapse, fetal distress, and Apgar at 5 min less than 7, stillbirth (newborn with EGA ≥ 28.0 weeks delivered with no signs of life, no breathing and no heart beat), and early neonatal death (live born infant with EGA ≥ 28.0 weeks who dies in the first 7 days of life).

### Ethics

Retrospective analysis of anonymised hospital records of the SMRU was approved and the need for individual consent was deemed unnecessary by the Oxford University Ethics Committee (OXTREC: 28 − 09) and the local community advisory board in Mae-Sot, Thailand (T-CAB-30/11/19), in adherence to the Declaration of Helsinki.

### Statistical analysis

To account for the non-randomised design of the study, we used propensity-score methods to reduce the effects of confounding. Using a logit model, propensity scores for the probability of being offered ECV were calculated for women who were offered ECV within a fixed window period such as it would be in a randomised controlled trial. Irrespective of fetal presentation prior to 35 weeks, if women attended ANC with breech presentation in the 35–37 week window, they were included, unless ECV was contraindicated. The 35–37 week window was chosen because the median week of all ECV at SMRU was 36 weeks and gestation at birth was 39, not 40 weeks. The first occurrence of breech in this window was the index case for propensity score analysis. Women were split into five blocks with similar propensities of being offered ECV. In theory, the difference between risk of breech at birth in women who were offered ECV and those who were not should be unbiased within each block of similar propensity scores. Balance of the propensity score across those who received ECV and those who did not was checked visually. The propensity scores, or predicted probabilities of being offered ECV, were used to calculate inverse probability weights which were normalised to sum to one. To check that the propensity scores were properly specified, balance of each covariate was checked using t-tests within each block and by computing the standardised differences of the covariates. Covariates for specification of propensity scores included year of ECV, placental position (posterior or not), timing of first ANC visit (first ANC in trimester 3 yes/no), smoker or not, pre-eclampsia, BMI at time of ECV (continuous), parity, and marginalized status (refugee or migrant).

Associations between ECV and breech at birth were then estimated using weighted logistic regression models. We also fitted a model that included the propensity score as a covariate.

For all women offered ECV with breech presentation from 28 weeks onward, the difference in proportion of breech at birth was reported and association with success or not was explored using logistic regression for maternal and neonatal outcomes. The selection of factors to include in a multivariable model with breech presentation at birth as the dependent variable were guided by univariate associations with successful or unsuccessful ECV. Model fits were confirmed using the Hosmer–Lemeshow goodness-of-fit test.

All analysis was done using Stata v17.0 (College Station, USA).

## Results

**Fig. 1:**
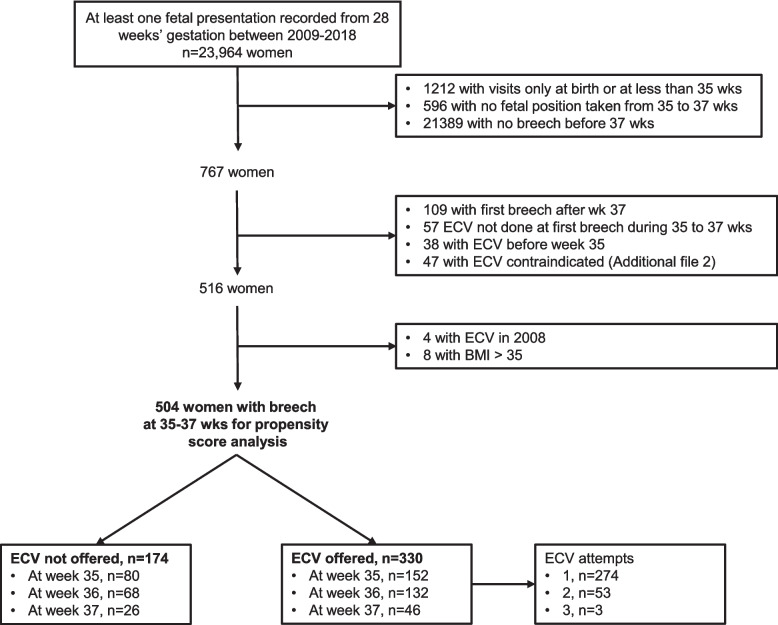
Flow chart of women included in the propensity score analysis. (wks = weeks, ECV = external cephalic version, BMI = body mass index)

Of 23,694 women with fetal position recorded from 28 weeks and with a singleton pregnancy outcome, most were excluded due to no fetal breech position (*n* = 21,389) (Fig. 1). Propensity scores were calculated for 504 women with breech presentation in the 35–37 weeks window. There were 330 women within this window who had ECV offered (1–3 attempts) and 174 for whom ECV was not offered (Table 1). Among women who had ECV offered, just under half (48.8%, *n* = 161) resulted in turning the baby. The majority of women (83.0%, *n* = 274) had only one ECV attempt, 53 women had two attempts (16.1%) and three women had three attempts (0.91%).

**Table 1:** Characteristics of women included in the propensity score analysis with breech at 35–37 weeks’ gestation

	Total Propensity cohort	ECV not offered	ECV offered		
**Maternal Characteristics**			Total ECV	Successful ECV	Unsuccessful ECV
N women	504	174 (34.5%)	330 (65.5%)	161/330 (48.8%)	169/330 (51.2%)
Median maternal age (p25, p75), yrs	26.0 (21.0, 33.0)	27.5 (21.0, 34.0)	26.0 (21.0, 32.0)	28.0 (23.0, 34.0)	24.0 (21.0, 31.0)
Parity, n (%)					
0	184 (36.5%)	65 (37.4%)	119 (36.1%)	36 (22.4%)	83 (49.1%)
1	115 (22.8%)	32 (18.4%)	83 (25.2%)	41 (25.5%)	42 (24.9%)
2 +	205 (40.7%)	77 (44.3%)	128 (38.8%)	84 (52.2%)	44 (26.0%)
Refugee (not migrant), n (%)	206 (40.9%)	84 (48.3%)	122 (37.0%)	62 (38.5%)	60 (35.5%)
Literate, n (%)	260/426 (61.0%)	75/132 (56.8%)	185/294 (62.9%)	81/139 (58.3%)	104/155 (67.1%)
Smoker, n (%)	87 (17.3%)	33 (19.0%)	54 (16.4%)	32 (19.9%)	22 (13.0%)
Median BMI at time of ECV (p25, p75)	24.7 (22.8, 26.8)	24.4 (22.8, 26.3)	24.8 (22.8, 27.1)	24.4 (22.7, 26.8)	25.2 (23.0, 27.8)
Year of ECV					
2008–2010	120 (23.8%)	57 (32.8%)	63 (19.1%)	34 (21.1%)	29 (17.2%)
2011–2012	97 (19.2%)	36 (20.7%)	61 (18.5%)	30 (18.6%)	31 (18.3%)
2013–2014	92 (18.3%)	23 (13.2%)	69 (20.9%)	37 (23.0%)	32 (18.9%)
2015–2016	117 (23.2%)	36 (20.7%)	81 (24.5%)	37 (23.0%)	44 (26.0%)
2017–2018	78 (15.5%)	22 (12.6%)	56 (17.0%)	23 (14.3%)	33 (19.5%)
First ANC presentation, n (%)					
Trimester 1	232 (46.0%)	85 (48.9%)	147 (44.5%)	69 (42.9%)	78 (46.2%)
Trimester 2	190 (37.7%)	54 (31.0%)	136 (41.2%)	66 (41.0%)	70 (41.4%)
Trimester 3	82 (16.3%)	35 (20.1%)	47 (14.2%)	26 (16.1%)	21 (12.4%)
Median EGA at time of ECV (p25, p75)	36.0 (35.0, 36.0)	Not applicable	36.0 (35.0, 36.0)	36.0 (35.0, 36.0)	35.0 (35.0, 36.0)
Posterior placental position, n (%)	280/501 (55.9%)	106/172 (61.6%)	174/329 (52.9%)	73/160 (45.6%)	101/169 (59.8%)
ECV related complications	*Not applicable*	*Not applicable*	17 (5.2%)*	4 (2.5%)	13 (7.7%)

*ECV* External cephalic version, *BMI* Body mass index, *ANC* Antenatal care, *p25 *25th centile, *p75* 75th centile, *yrs* years

^*^Including 3 maternal requests to stop and *n* = 14 fetal distress (either bradycardia or tachycardia)

There were 47 women with ECV contraindications (Fig. 1): 26 were absolute (established labour *n* = 10, preterm rupture of membranes *n* = 6, or ‘other’ *n* = 10) and 21 were relative (previous caesarean section n = 19, fetal death in utero *n* = 2). The other group included known poor obstetric history (*n* = 3), large ovarian cyst (*n* = 1), polyhdramnios (*n* = 1), placenta praevia (*n* = 1), antepartum haemorrhage (*n* = 1), intellectual disability (*n* = 1), bicorunate uterus (*n* = 1), and known congenital abnormality (*n* = 1). The outcomes of these 47 cases were summarised (Additional file 2) and not analysed further.

Propensity scores, or the predicted probabilities of ECV, were calculated and their distribution showed adequate balance between groups (Additional file 3). Covariate balance was checked by inspection of their standardised mean differences before and after weighting the sample using the propensity scores (Additional file 4).

A regression model with inverse probability weighting according to the propensity score showed no significant association between ECV (offered or not offered) and breech presentation at birth (Odds Ratio (OR) 1.20, 95% confidence interval (95% CI) 0.82 to 1.77). Findings were similar when adjusted for likely confounders, including EGA at ECV, year of ECV, placental position, parity, late ANC presenter, maternal smoking, pre-eclampsia, refugee or migrant, and BMI at time of ECV (adjusted OR 1.23, 95% CI 0.82 to 1.83). The same conclusion resulted when propensity scores were included as a covariate in the adjusted model (aOR 1.21, 95% CI 0.81 to 1.80) and for a multivariable model without propensity score weighting (data not shown).

In the propensity cohort of women who had ECV attempted, 50.9% (168/330) were breech at birth compared with 47.7% (83/174) in women with no ECV attempt (Table 2). In other words, among women with a breech presentation at 35–37 weeks and no ECV attempt, the rate of spontaneous version from breech to cephalic was 52.3% (91/174). The proportion of caesarean for breech at birth was 22.4% (74/330) in ECV attempted (*n* = 1 in the successful ECV group, unsuccessful *n* = 73) and 20.1% (35/174) in ECV not attempted (*p* = 0.549).

**Table 2:** Maternal and neonatal outcomes in the propensity score analysis cohort with breech at 35–37 weeks’ gestation

	Total Propensity cohort	ECV not offered	ECV offered		
**Maternal Characteristics**			Total ECV	Successful	Unsuccessful
N women	504	174 (34.5%)	330 (65.5%)	161/330 (48.8%)	169/330 (51.2%)
Pre-eclampsia, n (%)	16 (3.2%)	5 (2.9%)	11 (3.3%)	2 (1.2%)	9 (5.3%)
Birth outcomes					
Median Birthweight^$^ (p25, p75), g	2900 (2640, 3155)	2860 (2580, 3105)	2920 (2655, 3170)	3025 (2750, 3260)	2800 (2560, 3060)
Median EGA at birth (p25, p75), wks	39.3 (38.4, 40.2)	39.2 (38.3, 40.2)	39.4 (38.5, 40.1)	39.6 (39.2, 40.4)	39.0 (38.2, 39.6)
Male, n (%)	210 (41.7%)	75 (43.1%)	135 (40.9%)	69 (42.9%)	66 (39.1%)
Adverse perinatal outcome^*#*^, n (%)	30 (6.0%)	13 (7.5%)	17 (5.2%)	3 (1.9%)	14 (8.3%)
Cord prolapse	1 (0.20%)	1 (0.57%)	0	0	0
Fetal distress	14/309 (4.5%)	5/100 (5.0%)	9/209 (4.3%)	1/128 (0.8%)	8/81 (9.8%)
Apgar at 5 min < 7	11/423 (2.6%)	4/137 (2.9%)	7/286 (2.5%)	3/139 (2.2%)	4/147 (2.7%)
Stillbirth	5 (1.0%)	2 (1.1%)	3 (0.9%)	0 (0.0%)	3 (1.8%)
Early neonatal death	6 (1.2%)	2 (1.1%)	4 (2.1%)	1 (0.6%)	3 (1.8%)
Congenital abnormality, n (%)	17 (3.4%)	8 (4.6%)	9 (2.7%)	3 (1.9%)	6 (3.6%)
Preterm birth, n (%)	24 (4.8%)	11 (6.3%)	13 (3.9%)	0 (0.0%)	13 (7.7%)
Placental abruption, n (%)	1 (0.20%)	0	1 (0.30%)	1 (0.62%)	0
Place of birth, n (%)					
Hospital	139 (27.6%)	48 (27.6%)	91 (27.6%)	14 (8.7%)	77 (45.6%)
Home	56 (11.1%)	26 (14.9%)	30 (9.1%)	19 (11.8%)	11 (6.5%)
SMRU	309 (61.3%)	100 (57.5%)	209 (63.3%)	128 (79.5%)	81 (47.9%)
Breech at birth, n (%)	251 (49.8%)	83 (47.7%)	168 (50.9%)	9 (5.6%)	159 (94.1%)
Type of birth, n (%)					
Vaginal Breech	142 (28.2%)	48 (27.6%)	94 (28.5%)	8 (5.0%)	86 (50.9%)
Caesarean Breech	109 (21.6%)	35 (20.1%)	74 (22.4%)	1 (0.6%)	73 (43.2%)
Vaginal Cephalic Birth/Assisted†	236 (46.8%)	87 (50.0%)	149 (45.2%)	140 (87.0%)	9 (5.3%)
Caesarean Cephalic	17 (3.4%)	4 (2.3%)	13 (3.9%)	12 (7.5%)	1 (0.6%)

*EGA* Estimated gestational age, *AMRU* Shoklo Malaria Research Unit, *p25* 25th centile, *p75* 75th centile, *wks* weeks, *g* grams

^$^Among 459 with birthweight measured in first 72 h

^#^more than one adverse event possible e.g. fetal distress and Apgar < 7 at 5 min

^†^ *n* = 9 assisted (*n* = 8 vacuum extraction and n = 1 forceps) in Successful ECV (*n* = 2) and ECV not offered (*n* = 7, including forceps)

### Complications at the time of ECV in the propensity cohort

Complications at the time of ECV occurred in 5.2% (17/330) of women and were transient and resolved spontaneously or with supportive measures (Table 1). Three were maternal requests to stop the procedure and 14 were due to fetal distress, predominantly bradycardia (*n* = 13 women), and one case was tachycardia.

### Complications in labour in the propensity cohort

There were 37 adverse perinatal outcomes in 30 neonates: 7.5% (13/174) when ECV was not offered and 5.2% (17/340) when ECV was attempted (Table 2). There was one case of cord prolapse, 0.6% (1/174) in a footling breech in the ECV not offered group, and the neonate survived after an emergency caesarean breech birth (Additional file 5, row 27). There was one partial placental abruption detected after delivery of a retroplacental blood clot with the neonate discharged home on day 6, in the ECV (success) group (Additional file 5, row 16).

Adverse perinatal outcomes (5.2% (17/330) vs 7.5% (13/174), *p* = 0.295), preterm birth (3.9% (13/330) vs 6.3% (11/174), *p* = 0.232) and congenital abnormalities (3.0% (10/330) vs 4.6% (8/174), *p* = 0.367) were similar whether ECV was offered or not (Table 2). Stillbirth and early neonatal deaths were also similar (*p* = 0.889 and 0.951, respectively).

Each case of adverse perinatal outcome was reviewed and four were considered direct complications of asphyxia due to vaginal breech birth and three of these four neonates were born at home with unskilled birth attendants while the fourth was a clinic birth complicated by pre-eclampsia (Additional file 5). Twenty percent (6/30) had congenital abnormalities, none of whom survived: one stillbirth, four neonatal deaths and one who died on day 17 after birth (Additional file 5).

### Successful versus unsuccessful ECV

Of 23,694 women with fetal position recorded from 28 weeks and with a singleton pregnancy outcome, 537 women were documented to have at least one ECV attempt, with the earliest ECV offered at 32 weeks and the latest at 40 weeks gestation. After a failed first attempt, there were 83 women with a second attempt (34 successful (41.0%)) and 10 women with a total of three ECV attempts (2 successful (20.0%)), for a total of 640 ECV attempts (Table 3). Notably, approximately half of women were in the ECV successful group (50.8%) and half in the unsuccessful ECV group (49.2%), i.e., the ECV success rate in this cohort was 50.8% (273/537). Women in the successful ECV group were older, on average, by about 3 years, had higher parity, were less literate, with lower BMI, and a higher proportion of smokers and posterior placental location, compared to women in the unsuccessful ECV group (Table 3).

**Table 3:** Characteristics of women with ECV attempted

		**Total**	**Successful ECV**	**Unsuccessful ECV**	***p*-value**
N women		537	273 (50.8%)	264 (49.2%)	
Maternal Characteristics					
Median maternal age (p25, p75), yrs		26.0 (21.0, 32.0)	28.0 (23.0, 33.0)	25.0 (21.0, 32.0)	< 0.001
Parity, n (%)	0	202 (37.6%)	67 (24.5%)	135 (51.1%)	< 0.001
	1	114 (21.2%)	61 (22.3%)	53 (20.1%)	
	2 +	221 (41.2%)	145 (53.1%)	76 (28.8%)	
Refugee (v. migrant status), n (%)		195 (36.3%)	100 (36.6%)	95 (36.0%)	0.88
Literate, n (%)		294/489 (60.1%)	131/244 (53.7%)	163/245 (66.5%)	0.004
Smoker, n (%)		79 (14.7%)	50 (18.3%)	29 (11.0%)	0.017
Median BMI at time of first ECV (p25, p75)		24.8 (22.7, 27.0)	24.2 (22.5, 26.4)	25.4 (23.0, 27.7)	0.004
Year of ECV	2008–2010	83 (15.5%)	47 (17.2%)	36 (13.6%)	0.57
	2011–2012	91 (16.9%)	46 (16.8%)	45 (17.0%)	
	2013–2014	136 (25.3%)	72 (26.4%)	64 (24.2%)	
	2015–2016	139 (25.9%)	69 (25.3%)	70 (26.5%)	
	2017–2018	88 (16.4%)	39 (14.3%)	49 (18.6%)	
First ANC presentation, n (%)	Trimester 1	229 (42.6%)	113 (41.4%)	116 (43.9%)	0.74
	Trimester 2	225 (41.9%)	115 (42.1%)	110 (41.7%)	
	Trimester 3	83 (15.5%)	45 (16.5%)	38 (14.4%)	
Median EGA at time of first ECV (p25, p75)		36.0 (35.0, 36.0)	36.0 (35.0, 36.0)	36.0 (35.0, 36.0)	0.51
Posterior placental position, n (%)		234 (46.3%)	136 (51.7%)	98 (40.5%)	0.012
ECV related complications*		25 (4.7%)	6 (2.2%)	19 (7.2%)	0.006
Number of ECV attempts	1	444 (82.7%)	237 (86.8%)	207 (78.4%)	0.017
	2	83 (15.5%)	34 (12.5%)	49 (18.6%)	
	3	10 (1.9%)	2 (0.7%)	8 (3.0%)	

*ECV* External cephalic version, *BMI* Body mass index, *ANC* Antenatal care, *EGA* Estimated gestational age, *p25* 25th centile, *p75* 75th centile, *yrs* years

^*^Including 3 maternal requests to stop, *n* = 22 fetal distress (either bradycardia or tachycardia)

The proportion with breech presentation at birth was 4.4% (12/273) and 90.9% (240/264), in the ECV successful and unsuccessful groups (*p* < 0.001, Table 4). The proportion of caesarean breech birth was 1.1% (3/273) in ECV successful compared to 40.5% (107/264) in the ECV unsuccessful group (*p* < 0.001). Adverse perinatal outcomes and preterm birth were significantly lower in the ECV successful group: 3.7% (10/273) vs 9.1% (24/264), *p* < 0.01 and 0.4% (1/273) vs 6.8% (18/264), *p* < 0.001.

**Table 4:** Maternal characteristics and birth outcomes with at least one ECV attempt

Maternal Characteristics	Total	Successful ECV	Unsuccessful ECV	*p*-value
N women	537	273 (50.8%)	264 (49.2%)	
Pre-eclampsia, n (%)	15 (2.8%)	3 (1.1%)	12 (4.5%)	0.015
Birth Outcomes				
Median Birthweight* (p25, p75), g	2935 (2670, 3200)	3020 (2750, 3380)	2830 (2570, 3070)	< 0.001
Median EGA at birth (p25, p75)	39.4 (38.5, 40.2)	39.6 (39.2, 40.4)	39.0 (38.2, 39.8)	< 0.001
Male, n (%)	241 (44.9%)	134 (49.1%)	107 (40.5%)	0.046
Adverse perinatal outcome^#^, n (%)	34 (6.3%)	10 (3.7%)	24 (9.1%)	0.010
Cord prolapse, n (%)	0	-	-	-
Fetal distress, n (%)	17/333 (5.1%)	6/210 (2.7%)	11/123 (8.9%)	0.015
Apgar at 5 min < 7, n (%)	15/443 (3.4%)	5/231 (2.2%)	10/212 (4.7%)	0.138
Stillbirth, n (%)	4 (0.7%)	0 (0.0%)	4 (1.5%)	0.041
Early neonatal death, n (%)	6/533 (1.1%)	2 (0.7%)	4/260 (1.5%)	0.049
Congenital abnormality, n (%)	16 (3.0%)	7 (2.6%)	9 (3.4%)	0.565
Preterm birth, n (%)	19 (3.5%)	1 (0.4%)	18 (6.8%)	< 0.001
Placental abruption, n (%)	1/500 (0.2%)	1/260 (0.4%)	0	1.00
Place of birth, n (%)				
Hospital	143 (26.6%)	26 (9.5%)	117 (44.3%)	< 0.001
Home	39 (7.3%)	26 (9.5%)	13 (4.9%)	
SMRU	355 (66.1%)	221 (81.0%)	134 (50.8%)	
Breech at birth, n (%)	252 (46.9%)	12 (4.4%)	240 (90.9%)	< 0.001
Type of birth, n (%)				
Vaginal Breech	142 (26.4%)	9 (3.3%)	133 (50.4%)	< 0.001
Caesarean Breech	110 (20.5%)	3 (1.1%)	107 (40.5%)**	< 0.001
Vaginal Cephalic Birth/Assisted†	262 (48.8%)	242 (88.6%)	20 (7.6%)	< 0.001
Caesarean Cephalic	23 (4.3%)	19 (7.0%)**	4 (1.5%)	0.002

*ECV* External cephalic version, *EGA* Estimated gestational age, *p25* 25th centile, *p75* 75th centile, *g* grams, *SMRU* Shoklo Malaria Research Unit

^#^more than one adverse event possible e.g., fetal distress and Apgar < 7 at 5 min

^†^*n* = 6 assisted (vacuum extraction) in Successful ECV group

^*^Among 502 with birthweight measured in first 72 h

^**^Emergency caesarean due to antepartum haemorrhage (*n* = 2), one 4 weeks 4 days after successful ECV and one 1 week after unsuccessful ECV; both neonates had Apgars 9, 10 at 1 and 5 min

Factors associated with breech presentation at birth were explored in a multivariable logistic regression model. The only factor significantly associated with breech presentation at birth was an unsuccessful ECV (Table 5).

**Table 5:** Multivariable model of factors associated with breech presentation at birth in women with ECV attempted (*n* = 458/537 women included)

	Adjusted Odds Ratio	(95% CI)		*p*
Unsuccessful ECV (vs. successful ECV)	207.0	89.5	478.6	< 0.001
BMI at time of ECV	0.99	0.90	1.10	0.899
Posterior placental position	1.33	0.63	2.79	0.454
Parity 0	Ref			-
Parity 1	0.76	0.30	1.94	0.562
Parity 2 +	0.82	0.34	1.97	0.658
Smoker	0.55	0.18	1.75	0.315
Pre-eclampsia	6.14	0.45	84.2	0.174
1 ECV attempt	Ref			-
2 attempts	2.28	0.73	7.09	0.155
3 attempts	3.01	0.02	565.9	0.680
Literate	0.60	0.27	1.34	0.213

*ECV* External cephalic version, *BMI* Body mass index, *CI* Confidence interval

## Discussion

This resource-limited cohort of women provided a unique opportunity to compare women who were offered ECV, to women with breech presentation in the same gestational age window, who were not offered ECV. Analysis by propensity score suggested that an ECV attempt at 35–37 weeks was not associated with a lower odds of breech at birth (adjusted OR 1.23, 95% CI 0.82 to 1.83). This was possibly due to a spontaneous version rate of breech to cephalic presentation of 52.3% in the group who were not offered ECV (91/174). These findings suggest that an improvement in success rates would be required for ECV to out-perform spontaneous version rates in this setting, for example by considering adjuvant measures such as regional analgesia [9].

The rate of spontaneous reversion to breech presentation after successful ECV was within the range reported in other studies [9]. Earlier trials reported a wide range in the differences and proportions of breech at birth in the ECV group and no ECV group: 3.3% (1/30) and 67.0% (20/30) in 1983 in South Africa [10]; 52.3% (162/310) and 51% (168/330) in 1985 in Zimbabwe [11]; 43.8% (39/89) and 74.4% (67/90) in 1989 in the Netherlands [13]; and 20.0% (6/30) and 93.3% (28/30) in 2012 in India [12]. As with previously published literature, when only women who were offered ECV were considered in the analysis, the procedure was associated with considerably improved maternal and neonatal outcomes when successful [24]. The proportion of breech at birth was significantly lower for women with successful ECV compared to unsuccessful ECV (4.4% vs. 90.9%, Table 4) in this cohort, and there were significantly fewer adverse pregnancy outcomes.

In the propensity cohort, the proportion of breech in labour who birthed vaginally was 28.2% (142/504), similar to 32.6% (383/568) in Norway [25] but lower than other low-income settings such as Southern Ethiopia with 82.6% (317/384) [26]. There were no breech related perinatal deaths in the Norway cohort, but in Southern Ethiopia intra-uterine fetal death occurred in 5.7% (*n* = 22) and death in the first 5 min after birth in 16.1% (*n* = 62) of infants. This compares with a total of five stillbirths (1.0%) and six early neonatal deaths (1.2%) in 504 pregnancies in the propensity cohort, which included both cephalic and breech presentations. Among clinic-based births the majority of adverse outcomes were associated with lethal congenital abnormalities. Three of the deaths were suspected to be a result of breech birth asphyxia which occurred at home with non-skilled birth attendants. SMRU maintains skilled birth attendant breech birth skills using the Advanced Life Support in Obstetrics training [22].

### Strengths of this study

As far as we are aware, this was the first time propensity score analysis has been utilised in a population cohort to explore the impact of ECV on fetal breech presentation in labour. In this setting with sites spread over 150 km it was not possible to always have a doctor with ECV skills available when women were in the gestational window for ECV thereby providing an opportunity for a control group of women who were not offered ECV. Another strength was the use of propensity score analysis to reduce the effects of confounding due to the observational study design. The data presented from this resource-limited setting where ultrasound by local sonographers is routinely available suggests ECV is safe with a low rate of procedural complications [24], but access to ultrasound may not be possible in other LIC. ECV was conducted with a car on standby to take women to hospital if needed but the car was never required for any of the ECVs to date.

### Limitations

The exact reason why ECV was not done was not individually recorded for every woman prospectively but assigned retrospectively by record review. It was not possible to know exactly for every case if the doctor was present or not nor how many women did not turn up for expected ECV appointments although these points were noted ad hoc. Nonetheless the risk of bias was minimised by independent extraction by two authors. There was omission of systematic recording of certain factors that may be important including amniotic fluid index (AFI), z-score for estimated fetal weight, use of uterine relaxants and skill level of the physician involved [6].

### Interpretation

For each two-year block there were 83 to 139 ECV procedures done (Table 3), only 3 to 6 on average per month but not all by the same practitioner. It can be difficult to improve success when there are only a small number of cases to manage and 3 birth units (at the time of this cohort) spaced over 150 km of the Thailand-Myanmar border. However in a series of 290 consecutive breech cases, Kim et al., suggested that inexperienced physicians can experience success with multipara and by accumulating this experience, they will soon manage nullipara cases successfully [27]. In this setting the problem of no physician skilled in ECV could potentially be solved if senior SBA were taught the procedure as they can provide services seven days per week i.e. task shifting.

Although two decades have passed since the Term Breech Trial, the impacts remain with caesarean section dominating as mode of delivery for women with breech presentation [5, 28]. Re-evaluation of the findings and reflection have permitted a more nuanced approach for breech presentation with guidance recommending discussion with women to explain the choices and risks of external cephalic version (ECV), planned vaginal birth or planned caesarean section [9, 29–32].

At SMRU, the ultrasound staff are trained in gestational age assessment, not in detection of congenital abnormalities, although in some cases e.g., microcephaly and ventriculomegaly, were observed during dating scans. That one in five perinatal adverse events occurred with congenital abnormalities is consistent with the global literature on breech [33]. In high income countries where care for congenital abnormalities is available, caesarean section may be the preferred mode of birth or termination may be offered earlier in gestation. In resource-limited settings and conflict zones such as the Thailand-Myanmar border, safe termination of pregnancy, corrective paediatric surgery, assistive devices, and social supports to improve quality of life for people living with congenital abnormalities are often unavailable. Under these circumstances, the safest option for maternal health and the most compassionate option for the infant with severe congenital abnormalities, may be vaginal breech birth and neonatal palliative care. A caesarean delivery for a baby who will not survive jeopardizes the mother’s health and future ability to build a family in settings where a second or third caesarean delivery may not be safe or available.

Among women with ECV attempted, half resulted in a turned baby (50.8%, Table 3). This suggests that strategies to improve the success rate of ECV are needed. Adjuvant measures such as regional analgesia could be considered [9]. Task shifting, i.e., the practice of moving tasks from highly qualified health workers to those with less training and qualifications could be another method to improve access to this specific health service. A proof of this concept has been task shifting caesarean section, by non-physician clinicians in Africa [34]. Caesarean section is considerably more difficult than ECV. The success of ECV can be improved by standardising guidelines for recognition and preferably ultrasound confirmation of breech presentation at 36 weeks and above, selecting suitable candidates for ECV, screening effectively for the risks and benefits of ECV, applying best practice at each ECV attempt, and implementing effective interpersonal team strategies to support women undergoing ECV [35]. Following this analysis SMRU created a standardised ECV data collection system to avoid the limitations reported here. We hope this analysis will encourage other settings to perform propensity score analyses which presents a more holistic picture of breech in late pregnancy and breech birth.

## Conclusion

Evidence comparing outcomes after ECV support its usage as maternal and perinatal results are substantially better in successful versions. However, propensity score analysis of attempted ECV at 35 to 37 weeks offers a different perspective suggesting no benefit or harm with respect to breech presentation at birth given the balance between the spontaneous version rate of 52% and ECV success of 51%. Women need supportive counselling on the risks and benefits of vaginal breech birth, ECV and caesarean section. Sonographers can support detection and correct application of ECV so it needs to be accessible in LIC. Supporting practitioners to optimise ECV success has the potential to reduce maternal and perinatal morbidity and mortality in this and other resource-limited settings.

## Supplementary Information

Additional file 1.

Additional file 2.

Additional file 3.

Additional file 4.

Additional file 5.

## Data Availability

Data of the SMRU cannot be shared publicly because this is a population of undocumented refugees and migrants and we do not have their permission to share their data. Anonymised data are available from the Mahidol-Oxford Research Unit Institutional Data Access Committee upon reasonable request from researchers who meet the criteria for access to confidential data (contact Rita Chanviriyavuth, email rita@tropmedres.ac).

## Abbreviations

ANC: Antenatal care
BMI: Body mass index
CI: Confidence interval
ECV: External cephalic version
EGA: Estimated gestational age
LIC: Low-income countries
OR: Odds Ratio
aOR: Adjusted Odds Ratio
RCOG: Royal College of Obstetricians and Gynaecologists
SBA: Skilled Birth Attendant
SMRU: Shoklo Malaria Research Unit
