# Supplementary material for: Effect of external cephalic version in a resource-limited setting on the Thailand-Myanmar border: a retrospective cohort with propensity score analysis
Source: BMC Pregnancy Childbirth. 2026 Mar 12;26:433. doi: 10.1186/s12884-026-08917-5 (PMC13094156; doi:10.1186/s12884-026-08917-5)

Additional file 1 for:

**Effect of external cephalic version in a resource-limited setting on the Thailand-Myanmar border: a retrospective cohort with propensity score analysis**

Nay Win Tun, Nienke Vonk, Aung Myat Min, Mary Ellen Gilder, Gabie Hoogenboom, Lay Lay Wah, Wah Say, François Nosten, Marcus J. Rijken, Rose McGready, Sue J Lee

# **Additional File 1.** SMRU ECV Procedure and Obstetric Manual (4th Edition) ECV Protocol


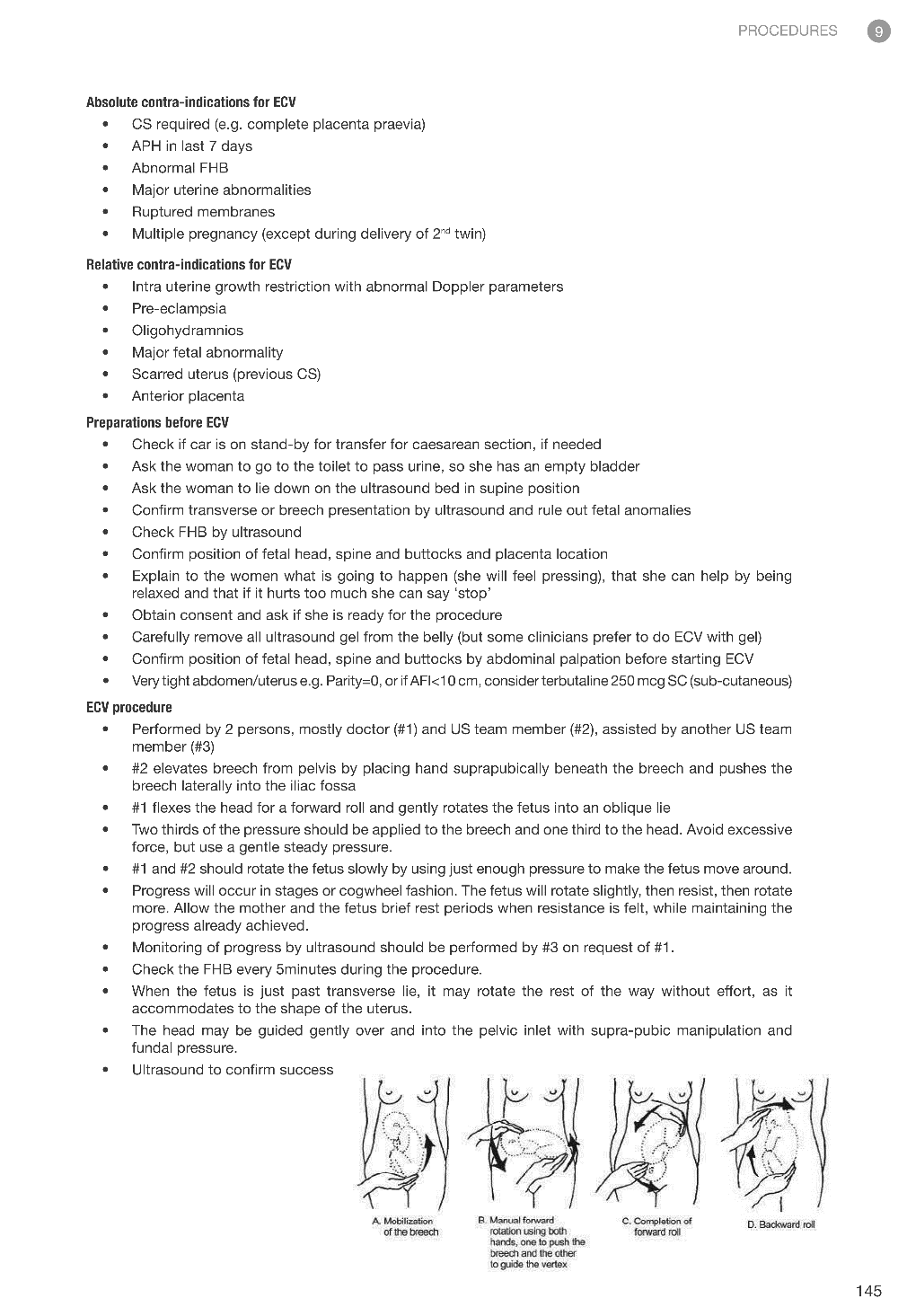


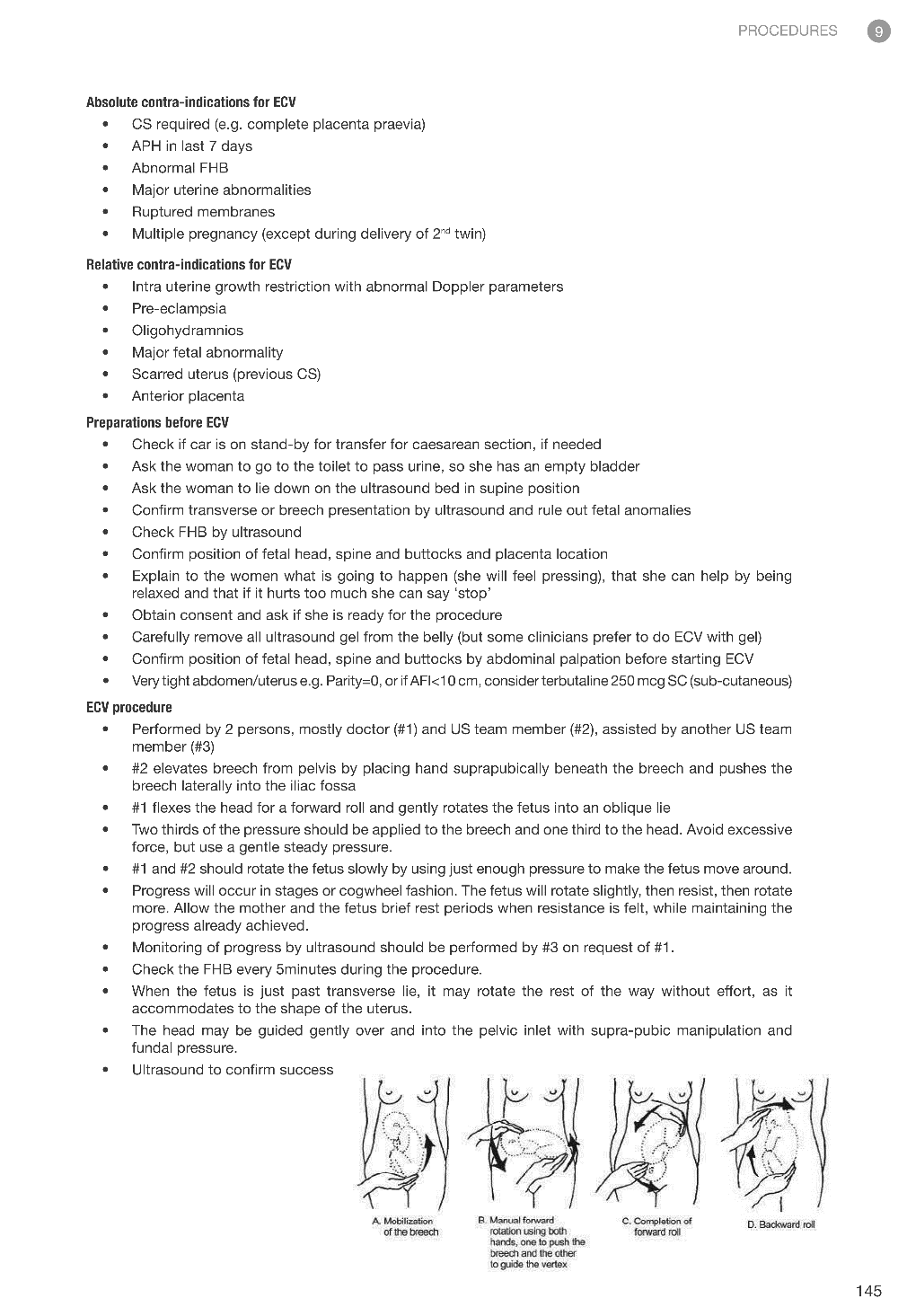

Supplement: Supplementary file 1 — Additional file 1. [file 12884_2026_8917_MOESM1_ESM.docx]
