# Supplementary material for: Effect of external cephalic version in a resource-limited setting on the Thailand-Myanmar border: a retrospective cohort with propensity score analysis
Source: BMC Pregnancy Childbirth. 2026 Mar 12;26:433. doi: 10.1186/s12884-026-08917-5 (PMC13094156; doi:10.1186/s12884-026-08917-5)
Supplement: Supplementary file 2 — Additional file 2. [file 12884_2026_8917_MOESM2_ESM.docx]

Additional file 2 for:

**Effect of external cephalic version in a resource-limited setting on the Thailand-Myanmar border: a retrospective cohort with propensity score analysis**

Nay Win Tun, Nienke Vonk, Aung Myat Min, Mary Ellen Gilder, Gabie Hoogenboom, Lay Lay Wah, Wah Say, François Nosten, Marcus J. Rijken, Rose McGready, Sue J Lee

**Additional file 2.** Birth outcomes of women with contraindicated ECV

|  | n (%)* |
| --- | --- |
| N women | 47 |
| **Birth outcomes** |  |
| Median Birthweight (p25, p75), g | 2610 (2300, 3100) |
| Median EGA at birth (p25, p75) | 37.3 (36.1, 38.5) |
| Male, n (%) | 23 (48.9%) |
| Pre-eclampsia, n (%) | 2 (4.26%) |
| Apgar at 5 min < 7, n (%) | 2/41 (4.88%) |
| Stillbirth, n (%) | 3 (6.38%) |
| Early Neonatal death, n (%) | 4 (8.51%) |
| Congenital abnormality, n (%) | 3 (6.38%) |
| Preterm birth, n (%) | 16 (34.0%) |
| Place of birth |  |
| Hospital | 30 (63.8%) |
| Home | 1 (2.13%) |
| SMRU | 16 (34.0%) |
| Type of delivery |  |
| Caesarean Breech Birth, n (%) | 13 (27.7%) |
| Vaginal Breech Birth, n (%) | 16 (34.0%) |
| Cephalic Caesarean Birth, n (%) | 16 (34.0%) |
| Cephalic vaginal Birth/Assisted, n (%) | 2 (4.26%) |
| ** Unless otherwise stated* |  |

*ECV = external cephalic version, EGA = estimated gestational age, SMRU = Shoklo Malaria Research Unit*
