# Supplementary material for: Effect of external cephalic version in a resource-limited setting on the Thailand-Myanmar border: a retrospective cohort with propensity score analysis
Source: BMC Pregnancy Childbirth. 2026 Mar 12;26:433. doi: 10.1186/s12884-026-08917-5 (PMC13094156; doi:10.1186/s12884-026-08917-5)
Supplement: Supplementary file 3 — Additional file 3. [file 12884_2026_8917_MOESM3_ESM.docx]

Additional file 3 for:

**Effect of external cephalic version in a resource-limited setting on the Thailand-Myanmar border: a retrospective cohort with propensity score analysis**

Nay Win Tun, Nienke Vonk, Aung Myat Min, Mary Ellen Gilder, Gabie Hoogenboom, Lay Lay Wah, Wah Say, François Nosten, Marcus J. Rijken, Rose McGready, Sue J Lee

**Additional file 3****.** Distribution of propensity scores among women with ECV offered and ECV not offered.
