# Supplementary material for: Effect of external cephalic version in a resource-limited setting on the Thailand-Myanmar border: a retrospective cohort with propensity score analysis
Source: BMC Pregnancy Childbirth. 2026 Mar 12;26:433. doi: 10.1186/s12884-026-08917-5 (PMC13094156; doi:10.1186/s12884-026-08917-5)
Supplement: Supplementary file 5 — Additional file 5. [file 12884_2026_8917_MOESM5_ESM.docx]

Additional file 5 for:

**Effect of external cephalic version in a resource-limited setting on the Thailand-Myanmar border: a retrospective cohort with propensity score analysis**

Nay Win Tun, Nienke Vonk, Aung Myat Min, Mary Ellen Gilder, Gabie Hoogenboom, Lay Lay Wah, Wah Say, François Nosten, Marcus J. Rijken, Rose McGready, Sue J Lee

# **Additional File 5.** Detailed view of adverse perinatal outcomes of neonates in the propensity score cohort
